# 5-Fluorouracil-Encapsulated Films Using Exopolysaccharides from a Thermophilic Bacterium *Geobacillus* sp. WSUCF1 for Topical Drug Delivery

**DOI:** 10.3390/mi14051092

**Published:** 2023-05-22

**Authors:** Joseph M. Laubach, Rajesh K. Sani

**Affiliations:** 1 Department of Biomedical Engineering, South Dakota School of Mines and Technology, Rapid City, SD 57701, USA; 2 BuG ReMeDEE Consortium, South Dakota School of Mines and Technology, Rapid City, SD 57701, USA; 3 Department of Chemical and Biological Engineering, South Dakota School of Mines and Technology, Rapid City, SD 57701, USA

**Keywords:** 5-fluorouracil, melanoma, exopolysaccharide, *Geobacillus* sp., polymer films, topical drug delivery

## Abstract

Bacteria are capable of producing a specific type of biopolymer, termed exopolysaccharides (EPSs). EPSs from thermophile *Geobacillus* sp. strain WSUCF1 specifically can be assembled using cost-effective lignocellulosic biomass as the primary carbon substrate in lieu of traditional sugars. 5-fluorouracil (5-FU) is an FDA-approved, versatile chemotherapeutic that has yielded high efficacy against colon, rectum, and breast cancers. The present study investigates the feasibility of a 5% 5-fluorouracil film using thermophilic exopolysaccharides as the foundation in conjunction with a simple self-forming method. The drug-loaded film formulation was seen to be highly effective against A375 human malignant melanoma at its current concentration with viability of A375 dropping to 12% after six hours of treatment. A drug release profile revealed a slight burst release before it settled into an extended and maintained release of 5-FU. These initial findings provide evidence for the versatility of thermophilic exopolysaccharides produced from lignocellulosic biomass to act as a chemotherapeutic-delivering device and expand the overall applications of extremophilic EPSs.

## 1. Introduction

Microbial exopolysaccharides (EPSs) are long-chain carbohydrate polymers produced by microorganisms within the cell before they are pushed outside of the cell wall [1]. Phenotypically, EPSs can be excreted as a firm and tight capsule, or a sludge-like layer loosely attached to the microorganism. In nature, microbial EPSs are understood to play a defined role in the protection of the cell. Moreover, these biopolymers assist with adhesion of the microbes to a surface, development of biofilm structures, as well as contributes to cell-to-cell interactions [2,3].

A primary goal in drug delivery field is to improve the bioavailability of the delivery complexes by way of innovative formulation development. General concerns with developing a competent drug delivery vehicle involves a suitable biocompatibility as well as the potential for cytotoxicity of both the vehicle itself and degradation products [4,5,6]. EPSs have been reported to degrade into their respective monomers or short oligomers within the body through normal biological processes [6]. By utilizing EPSs, the body would be capable of naturally breaking down the biopolymer-derived drug delivery vehicle once the transfer of the drug is complete. Polysaccharide structures undergo degradation via the lysozyme, an antimicrobial combatant that cleaves the peptidoglycan portion of cell walls [7,8]. The strong similarities the biochemical properties of polysaccharides share with the extracellular matrices in humans help lysozymes recognize the carbohydrate polymers [9,10].

Thermophiles are a category of extremophilic bacteria that thrive in unusually high temperatures (>55 °C). Interestingly, thermophilic EPSs share in the host’s trait of delayed thermal degradation, subsequently making it quite valuable [11,12]. Although thermophiles are known to produce low yields of EPSs in comparison to mesophiles, the fermentation process of approximately 24 h or less makes thermophilic EPSs an asset to industrial applications when considering its quick turnaround time [1,12]. Thermophile *Geobacillus* sp. WSUCF1 possesses enzymes capable of the hydrolysis of cellulose and hemicellulose. The strain WSUCF1 can be cultured and grown employing lignocellulosic biomasses, such as unprocessed corn stover, without the need for expensive pretreatments. The strain produces a titer of 2.97 ± 0.45 g per liter of EPSs. Traditionally, fermentation of lignocellulosic materials needs a pretreatment procedure in order to increase the area of contact between material and enzymatic reagents from their otherwise uncooperative backbone [13,14,15]. The utilization of lignocellulosic biomass that does not require pretreatment as the primary carbon substrate can ultimately become valuable for industrial biomedical applications, where it is common to see the usage of pure sugar for the source of carbon during production of microbial EPSs [15].

Malignant melanoma is still the fatal form of skin cancer capable of spreading rapidly and possessing a high persistent capability [16]. Per the Center for Disease Control (CDC), in 2018, about 84,000 new melanoma cases were reported in the US alone. From 2009 until 2018, occurrences of malignant melanoma of the skin had increased by 1.2% per year on average [17]. As of today, there remains a lack of effective therapies for this skin disease owing to the drug resistance of melanoma [18].

5-fluorouracil (5-FU) is a general chemotherapeutic introduced in 1958 that exhibits a high efficacy against tumors seen in the likes of colon, rectum, and breast cancers [19,20,21]. It has been noted as to having a short half-life, said to be in the range of 8–20 min, and it shows rapid gastrointestinal absorption, where 80% of orally administered 5-FU has been metabolized within the body [19]. 5-FU displays severe toxicity in healthy cells and yields high digestive distress during oral administration, both hindering its application in cancer therapy [19,20]. In the present day, accurately designed formulations of 5-FU using biocompatible and biodegradable polysaccharide delivery vehicles have been investigated and employed for controlled delivery. Past research has stated that extremophilic EPS showed noticeable negative effects on the proliferation of malignant mammalian cells. It has been theorized that the EPSs exhibiting this effect may induce apoptosis of cancer cells by way of bonding to specific surface receptors [22,23].

Glutaraldehyde (GA) is a common crosslinking agent used in biomedical applications, including hydrogel and nanoparticle synthesis. It has been seen to be effective in the stabilization of biomaterials, and it is easily accessible, cost-effective, and has high aqueous solubility [24,25]. Its primary form is a colorless liquid that exhibits a rather pungent smell at room temperature. GA is a dialdehyde that contains two highly reactive aldehydic functional groups capable of forming covalent bonds with amines, thiols, phenols, hydroxyl, and imidazoles [24].

The primary focus of this study was to design and evaluate a low-cost, chemotherapeutic film derived from thermophilic exopolysaccharides for the purpose of topical drug delivery. The hypothesis of this research is based on the ability of polysaccharides to thicken, emulsify, encapsulate, swell, and flocculate to form the likes of gels, films, or membranes. In combination with this, EPSs contain many reactive functional groups which should lead to successful chemical adjustment and subsequent drug loading. Moreover, the hydrophilicity of EPSs, although tunable, leads to the theory that adequate release of the loaded drug will be achievable, thereby minimizing waste [26,27,28,29,30]. With this work, an economically competent novel drug delivery device that may offer extended drug release and enhanced efficiency with industrial potential could be offered. The films used here are formed using a basic crosslinking method and self-forming procedure where 5-FU was used as the drug of focus.

## 2. Materials and Methods

### 2.1. Materials

5-fluorouracil powder, glutaraldehyde as a 25% aqueous solution, molecular biology grade dimethyl sulfoxide (DMSO), and Whatman filter paper (22 µm pore size) were purchased from Sigma Aldrich (St. Louis, MO, USA). A375 human malignant melanoma epithelial cells (CRL-1619) and Dulbecco’s Modified Eagles Medium (30-2002) were purchased from ATCC (Manassas, VA, USA). Kits (CYQUANT LDH Cytotoxicity, Image-it™, and Vybrant™ MTT Cell Proliferation), Fetal bovine serum, reagents (Invitrogen ActinRed 555 ReadyProbes, NucBlue™ Fixed Cell Stain ReadyProbes™, and ProLong™ Gold antifade) were purchased from Thermo Fisher Scientific (Waltham, MA, USA). Further mentioned reagents, solvents, or chemicals were of molecular biology or HPLC grade.

### 2.2. Cell Culture

A375 human melanoma cell line (CRL-1619) was purchased from ATCC (Manassas, VA). These A375 cells were maintained in DMEM medium at 37 °C in a humidified (5% CO_2_, 95% air) atmosphere. The DMEM medium was amended with antibiotics (penicillin, 100 units/mL) and streptomycin, 50 units/mL) and fetal bovine serum (10%).

### 2.3. Precipitation and Extraction of the EPS

Media for WSUCF1 was made ready for use in a 1000 mL flask. To the flask, 6 g of minced corn stover, 20 g yeast extract, 3 g NaCl and 500 mL of distilled (DI) water were added. A separate flask was filled with 500 mL of DI water to eventually be autoclaved and added to the previous flask, which would bring the total volume of the media to 1000 mL [15]. The total volume of DI water was halved between two flasks to allow for head room within the flask during the autoclave process and to prevent boiling over. The reagents were mixed thoroughly using a magnetic stir bar for approximately 15 min. The solution was then adjusted to a pH of 7.0 before it was autoclaved at 121 °C for 20 min along with the other flask containing only 500 mL of DI water. When the media and water flasks had cooled, the two were combined into one flask, and the media were inoculated with WSUCF1. The flask was then situated in a shaker incubator adjusted to 60 °C for a duration of 24 h. Once the time point was reached, the bacterial culture was vacuum filtered through a Büchner funnel with the porous plate covered with a 0.22µm pore size Whatman paper filter intended to remove the biomass prior to the next step.

The bacterial cells from the culture are not required to proceed. Subsequently, they were isolated from the EPS-containing supernatant via centrifugation at 8000 rpm for 20 min in 500 mL centrifuge bottles. The cell precipitates were collected and disposed of while the supernatant was kept and used in the following steps. The thermophilic EPSs are protected from degradation in this optional step due to their thermophilic nature, while excess water is allowed to evaporate. To induce the precipitation of the crude EPS, a 1:1 ratio of absolute ethanol was poured into the remaining supernatant, manually stirred to provoke homogeneity, and stored in a −20 °C freezer overnight. It is worth noting that at this point, the ethanol/EPS container can be allowed to be stored at −20 °C for as long as is required. The authors have not noticed any detrimental effects to the collected EPSs by doing so. Once acquisition of the EPS was required, the ethanol/supernatant solution was centrifuged at a speed of 8000 rpm for 40 min [15]. The collected EPSs that were precipitated are capable of being stored at −20 °C without the need of a cryoprotectant.

### 2.4. Manufacturing the 5FU-Loaded Film

To a beaker, crude EPSs were added and mixed with distilled water using a magnetic stir bar to accomplish a 5% (*w*/*v*) concentration. Once homogenous, glycerol was introduced dropwise for the role of a plasticizer to a final concentration of 30% (g glycerol/g crude EPSs). 5-FU was prepared at a concentration of 5% (*w*/*w*, g 5-FU/g EPSs). 5-FU is sparingly soluble in water, but it is readily soluble in DMSO from 10–50 mg/mL. DMSO has been reported to inhibit cancer cell migration and proliferation [31]. 5-FU was dissolved in molecular biology grade DMSO and added to the film solution, followed by GA at a 10% concentration (*v*/*v*, 2 mL GA per 20 mL film solution). Stirring continued for approximately 30 min at 700 rpm. Into a PTFE evaporating dish, 20 mL of the EPS/5-FU solution was gently poured using serological pipets and allowed to cure within a dry heat oven for 48 h, or until excess water had evaporated [32].

### 2.5. Fourier-Transform Infrared Spectroscopy (FTIR)

FTIR spectra for an EPS film without the crosslinker and 5-FU, and an EPS/5FU film were documented by operating a Nicolet iS10 (Thermofisher, Waltham, MA, USA) FTIR spectrometer. The resolution for this specific spectrometer is 0.4 cm^−1^. Sections near the center of the film were carefully excised before a pair of heat-sterilized tweezers were employed to remove the small samples for analysis. Sixteen scans of the samples were captured from 4000 to 499 cm^−1^.

### 2.6. MTT Assay

An Invitrogen Vybrant™ MTT Cell Proliferation Assay Kit was implemented to document the results of A375 cells exposure to the EPS/5-FU film solution as well as a 5% (*w*/*w*, g 5-FU/g EPS) solution in DMSO. The protocol was executed as per the kit’s instructions. Assay data were acquired from an Epoch 2 microplate spectrophotometer (Biotek, Winooski, VT, USA). The data are reported as a cellular viability percentage. A cell suspension of 0.6 × 10^6^ cells/mL was prepared. A total of 500 µL of the cell suspension in fresh media was seeded into a 24-well plate using media free of phenol red. The cells were allowed an incubation time of 72 h. Positive controls that were intended to maintain 100% viability were not introduced to a sample of the film. Negative controls were meant to demonstrate complete or near-complete cell death, and therefore, they included 50 µL of 100% hydrogen peroxide. The assay was carried out in quadruplicate (*n* = 4). To provide exposure data, the cells were subjected to a 50 µL of the EPS/5-FU film-forming solution. This assay did not involve cured film sections due to previous experimentation, where the film gradually dissolved within cell media. Consequently, film residue was found to be coated across the well, even after the media change step that is required during the MTT assay protocol. This would inevitably lead to an inaccurate reading during the eventual analysis with the microplate reader.

### 2.7. LDH Assay

To add further evidence of the anticipated cytotoxicity to A375 cells, a lactate dehydrogenase (LDH) assay was conducted, with respect to the manufacturer’s guidelines, in a 96-well plate. An Epoch 2 microplate spectrophotometer (Biotek, Winooski, Vermont, USA) was utilized to acquire assay data. A suspension culture of 6000 cells per 100 µL of growth medium was prepared and added to each well. The plate was then allowed to incubate for 48 h prior to treatments. Chemical treatment was performed by introducing a 10 µL of either the EPS/5-FU complete film solution or a 5-FU solution in DMSO with a concentration equal to that required for the film solution. The experiment was performed in triplicate (*n* = 3). The absorbance was measured at two wavelengths (490 nm and 680 nm) using a microplate reader. To determine the amount of lactate dehydrogenase activity, the 680 nm value was treated as the background signal from the instrument and subtracted from the 490 nm value.

### 2.8. Fixed-Cell Fluorescence Imaging

ActinRed™ 555 ReadyProbes™ reagent, NucBlue™ Fixed Cell Stain ReadyProbes™ reagent, and an Image-it™ Fixation/Permeabilization kit (Life Technologies Corp., Eugene, OR, USA) were employed in this study to investigate the condition of the cellular structure when subjected to the 5-FU loaded film solution. A single round (22 mm) collagen-coated coverslip (BioCoat, Bedford, MA, USA) was placed into each well of a 6-well plate for the cells to adhere to. The seeding density of the cells was 0.3 × 10^6^ per well. The total volume of cells and media was 3 mL for each well. Cells were allowed 48 h within an incubator to reach confluency. Control wells where the cell line was not treated did not contain EPS/5-FU samples. Exposure experiments saw the addition of a 100 µL of EPS/5-FU film-forming solution added to the well. At 12 and 36 h after exposure, cells were immediately fixed using 4% formaldehyde in PBS. Once this was achieved, either the actin filaments or nucleus of the cells were marked for analysis with the utilization of epifluorescent stain. All the acquired images were not manipulated or altered prior to measuring corrected total cell fluorescence (CTCF). Photographs for both stains used the same imaging settings for that respective group. Images were viewed and photographed using an Olympus IX50 inverted fluorescence microscope.

### 2.9. HPLC Drug Release Profile

A newly casted EPS/5-FU film, measuring 3.1 inches in diameter, was placed in a 50 mL centrifuge tube and submerged in 50 mL of PBS (1X 0.01 M, pH 7.4). The tube was maintained at a temperature of 37 °C and simultaneously oscillated at 100 RPM to simulate human skin temperature and the natural movements of the body. Three separate samples (*n* = 3) of 1 mL volumes were pipetted from the receptacle at select junctures and preserved in a common refrigerator freezer until the total amount of samples were obtained at the conclusion of the examination. All unknown and known samples were sterile-filtered before injection into the HPLC. The mobile phase contained a mixture of 10% acetonitrile and 90% HPLC grade water. The pump was maintained at 0.6 mL/min, the detector was adjusted to 265 nm, and the column temperature was set to 25 °C. Samples were examined using a Prominence-i series LC-2030C Plus liquid chromatograph (Shimadzu, Kyoto, Japan). A 300 mm length × 7.8 mm inner diameter Aminex HPX-87H column was used.

With the intent of calculating the concentration of 5-FU found in the unknown samples, a series of 5-FU standards were recorded using the identical HPLC method. The values for area under the peak were graphed as the *y*-axis, while each respective known 5-FU amount represented the *x*-axis. The equation of the trendline for the series of points was used to calculate the sum of amikacin found in each unknown. Once an unknown sample was analyzed by the HPLC, it produced a value for the area under the peak that represents 5-FU. This value for area was plugged into the “y = mx + b” trendline equation as the “y” variable. By solving for “x,” the amount of 5-FU present in the unknown sample could be solved.

### 2.10. EPS/5FU Film Thickness

Utilization of a digital vernier caliper provided quantification of the thickness for a variety of newly casted EPS/5-FU films. The films were removed from their respective evaporating dish before the caliper measured the thickness directly over the film’s center. The specific instrument employed measures 0–150 mm and is accurate to 0.02 mm.

### 2.11. Swelling Ratio

A total of ten films were prepared: five without 5-FU and five containing 5-FU in the formulation. Once dry, they were then each weighed separately while still inside their respective PTFE evaporating dishes. Empty PTFE dishes that would encase the films were weighed and this weight was subtracted from the weight of the cured film in its dish. This was to ensure accurate weighing of a complete film. It is the belief that a well-crosslinked polymer film should exhibit a minimal degree of swelling. Swelling ratio (%) was calculated using the equation: (Final film weight—Initial film weight)/(Initial film weight) × 100

### 2.12. Statistical Analysis

Experimental data were examined in detail using OriginLab Corporation’s OriginPro 2022 graphing and analysis software. Experiments are noted within their respective sections as being performed in triplicate or quadruplicate. Statistically significant differences (*p* < 0.05) within experimental groups were calculated using one-way or two-way repeated measures ANOVA followed by a Tukey’s test post hoc.

## 3. Results and Discussion

### 3.1. Physical Characteristics of the EPS/5FU Films

**Figure 1:**
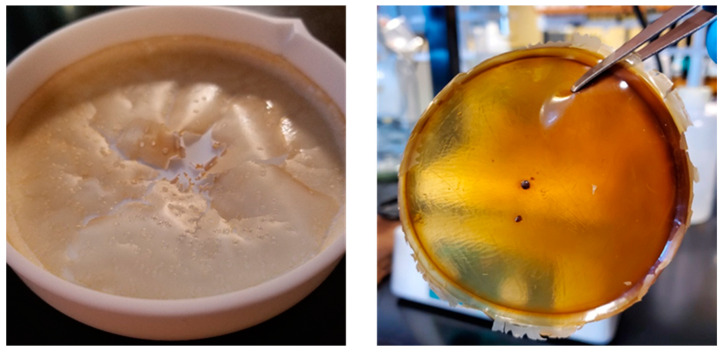
EPS film without the addition of GA crosslinker of 5-FU (**left**) and a 5% EPS/5-FU film containing GA and 5-FU (**right**).

In Figure 1, the picture on the left is of an EPS film using the same formula, but without the presence of GA and 5-FU. The picture on the right is a cured complete 5% EPS/5-FU film. A remarkable change in the physical characteristics of the film can be seen following the mentioned additions. The complete film exhibits characteristics of being semi-transparent, slightly adhesive, and a robust structure. The film was able to be peeled and removed from the PTFE dish with relative ease by using a pair of tweezers. Drug-delivering films and patches should be designed with enough flexibility to align with the curvature of the skin, but the mechanical properties should also not be weakened with the movement of the skin [33,34].

### 3.2. FTIR Analysis

**Figure 2:**
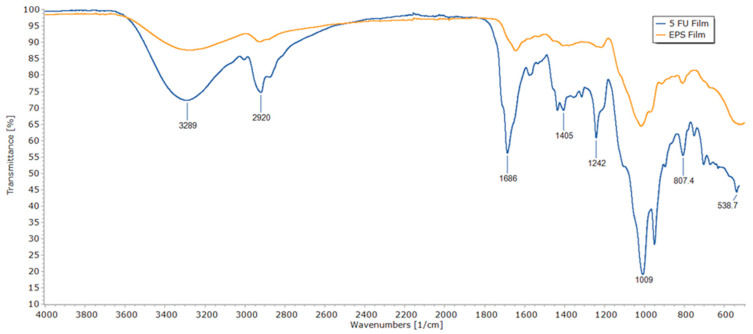
FTIR spectra of a basic EPS film without the presence of GA and 5-FU (top spectrum), and a 5% EPS/5-FU film (bottom spectrum).

FTIR spectra of an EPS film without GA and 5-FU and an EPS/5-FU film are shown in Figure 2. The wavenumber values for the peaks of significance for the EPS/5-FU film are indicated in the figure. A broad band that reaches its pinnacle at 3289 cm^–1^ is attributed to —NH stretching vibrations due to the presence of 5-FU. At 2920 cm^−1^, the EPS/5-FU film displays a C-H group stretching band. At approximately 1686 cm^−1^, the drug-loaded EPS film exhibits strong carbonyl stretching (C=O). The peak at 1242 cm^−1^ alludes to C-O stretching and the strong peak at 1009 cm^−1^ belongs to C-F stretching in the spectrum of 5-FU. This can be considered as evidence of encapsulation.

### 3.3. MTT Assay

With the intention of determining the metabolic activity of human malignant melanoma cells when exposed to the EPS film, the colorimetric MTT assay was performed. The values are reported in terms of cellular viability percentage. The motivation behind this study was to examine the potential efficacy of a film sourced from EPSs as a tool for topical chemotherapy against melanoma.

**Figure 3:**
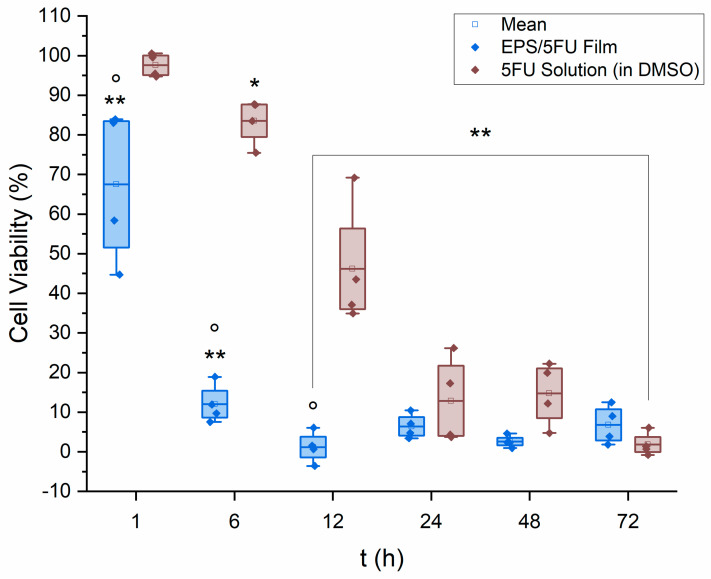
Viability of A375 cells exposed to the EPS/5-FU film-forming solution and a solution of 5-FU in DMSO of the same concentration used in the films (*n* = 4). Data were further evaluated for significance using a two-way repeated measures ANOVA as well as Tukey’s test post hoc. Versus untreated control: * *p* < 0.01, ** *p* < 0.0001. Versus free 5-fluorouracil in DMSO: ° *p* < 0.0001.

The results of the MTT cytotoxicity assay can be seen in Figure 3. The EPS/5-FU film solution was seen to be exponentially more effective within the first few hours of the assay. After 6 h of exposure, A375 cell viability was almost 7× higher on average for the 5-FU solution in comparison to the EPS/5-FU film solution. Cell media containing the chemical treatments were removed and fresh media were added to each well prior to the addition of MTT as to avoid potential interference. As mentioned previously, the potential apoptosis effect EPSs from extremophilic origin have on cancer cells may be assisting the chemotherapeutic against the melanoma cells.

In comparison, Ali et al. prepared solid lipid-based nanoparticles conjugated with 5-FU and examined them against B16F10 human melanoma cells. After 24 h of exposure, the authors noted less than 25% of the melanoma cells remained viable [35]. Using an induced melanoma animal model, Sahu et al. examined the capabilities of a 0.2% 5-FU (*w*/*v*) chitosan-based nanogel. The authors reported that 33% of mice within the treatment group experienced tumor growth. A separate exposure group within this study were treated with a 5% *w*/*v* 5-FU gel, similar to that used in industry, and reported a higher value of 61% tumor incidence [36]. Hao et al. utilized 5-FU loaded monomethoxy-poly (ethylene glycol)-polycaprolactone nanoparticles in conjunction with hyaluronic acid dissolvable microneedles to treat A375 human malignant melanoma cells in mouse models. The results of a 25-day in vivo study depicted successful and complete inhibition of the proliferation of A375 cells, without regrowth of the tumor [37]. Overall, the effectiveness of the film solution was seen as a time-dependent function, and not a question of dose-dependence, that lead to complete or near-complete cellular death.

The 5-FU potency or molar solution of each casted film calculates to be approximately 0.013 M, knowing the molecular weight of 5-FU is 130.078 g/mol. These results show the EPS/5-FU films may still be effective if the concentration of 5-FU or DMSO was reduced. Although the results here are positive, A375 cells exposed to a physical, fully cured EPS/5-FU film may have fared differently, as contact between the melanoma and the chemotherapeutic may not be as immediate or with immediate efficacy as seen here.

### 3.4. Lactate Dehydrogenase (LDH) Assay

**Figure 4:**
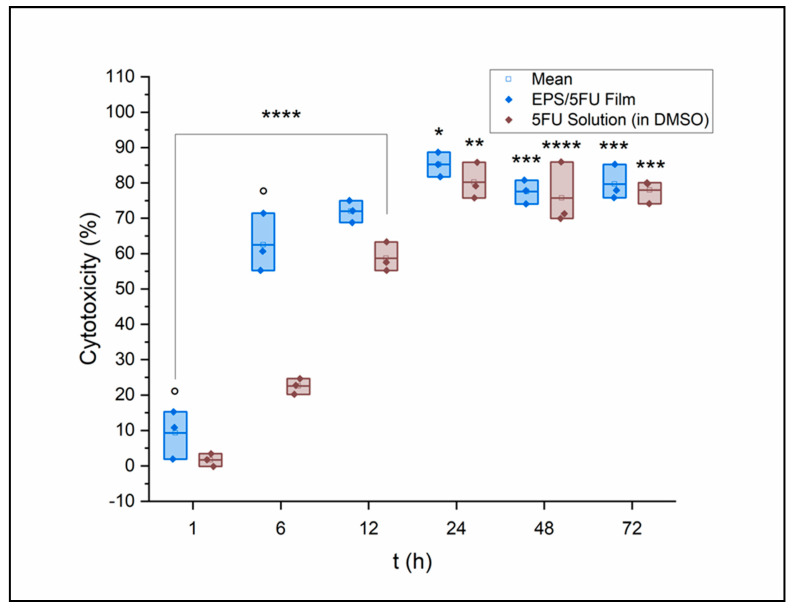
LDH assay results of A375 cells were treated with either the EPS/5-FU solution or a 5-FU solution in DMSO and compared (*n* = 3). Data were further analyzed and assessed for significance using a two-way repeated measures ANOVA and Tukey’s test post hoc. Versus the untreated control: * *p* < 0.001, ** *p* < 0.0001, *** *p* < 0.00001, **** *p* < 0.000001. Versus the free 5-fluorouracil in DMSO: ° *p* < 0.001.

Cytotoxicity values acquired from an LDH assay are seen in Figure 4. A375 cells were again treated with both the EPS/5-FU film solution and a 5-FU solution in DMSO of equal concentration to the film. The cell viability gap at the 6 h time point seen in the MTT assay is seen again after the LDH assay. Cytotoxicity at 6 h was measured via LDH release to be nearly 3× as great with EPS/5-FU exposure versus the 5-FU solution. During LDH assays, endogenous LDH activity in serum may cause the background signal during LDH assays. Cell media used in this assay utilized 5% FBS instead of the standard 10% to reduce the background signal that could possibly interfere with the results of the assay. However, a reduction in FBS may also compromise cell viability [38]. Cytotoxicity peaked for A375 cells at the 24 h point, where those treated with the film-forming solution reported an average cytotoxicity of 85%. Cytotoxicity values that may not have been as high as expected, or those that are seen to decrease after 24 h, can be attributed to the increasing acidity of the cell media over time as it incubates in the humid atmosphere of the CO_2_ incubator. A decrease in culture medium pH leads to less radiation absorption [39]. Culture medium is replaced as a part of the protocol for MTT assays, and therefore, this factor has a greater possibility of being avoided.

### 3.5. Fixed-Cell Fluorescence Imaging

**Figure 5:**
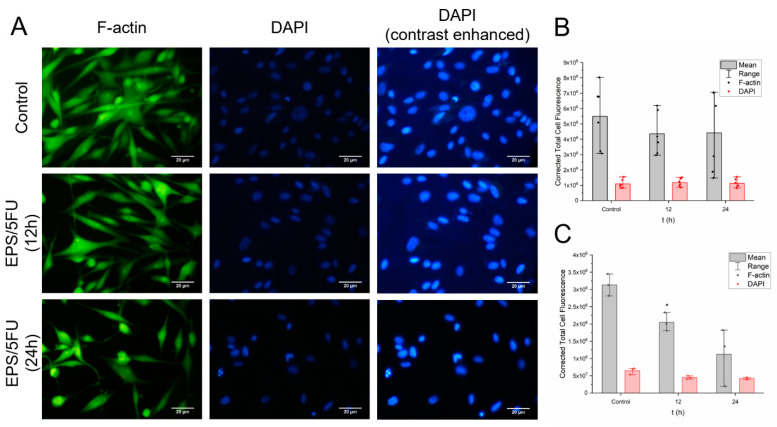
Results of A375 fixation fluorescence staining. (**A**) Photographs of A375 cells staining for F-actin and DAPI after treatments. (**B**) Corrected total cell fluorescence of individual cells experiencing and devoid of EPS/5-FU exposure (*n* = 6). (**C**) Corrected total cell fluorescence of cell groups with and without EPS/5-FU exposure (*n* = 6). Statistics were further analyzed and assessed for significance using a two-way repeated measures ANOVA and Tukey’s test post hoc. (**B**) Versus the untreated control *p* < 0.001. (**C**) Versus the untreated control * *p* < 0.001.

Figure 5a–c represent the data obtained for cell fluorescence intensity at 12 h and 24 h after A375 human malignant melanoma cells were subjected to the 5% EPS/5-FU film forming solution. Data seen in Figure 5b,c are illustrated as individual data points plus the mean. To acquire fluorescence intensity data, areas on the coverslip were chosen at random for analysis. Within these areas, six random individual cells from six different sectors of the photograph had their fluorescence intensity quantified with help from the software ImageJ. The data were then copied to a Microsoft Excel document and the formula mentioned below was implemented to calculate for corrected total cell fluorescence (CTCF):

Where CTCF = Integrated Density − (Area of selected cell × Mean fluorescence of background readings)

To perform a correspondent experiment to CTCF acquisition, four arbitrarily chosen locations on the individual coverslips, different from the locations used for CTCF calculations, were selected for cell counting. From these locations, A375 cells that were visible within the frame of view were counted using the DAPI staining. This method was performed for each exposure as well as the untreated group. The purpose of this parallel experiment was to obtain further evidence for the loss of viable A375 cells after treatment with the 5-FU/EPS film solution, like what was seen following the MTT and LDH assays. Furthermore, cell counting would provide a beneficial supplementation to the data acquired for CTCF. The results are displayed in Table 1. From the areas of the coverslips that were selected, a noticeable decrease in cell presence was seen.

This experiment was performed using a film solution, because it is believed that performing this in vitro assay using portions of a solid film could potentially lead to inaccurate results. As stated previously in this manuscript, performing similar experiments with a cured film previously resulted in film residue being left behind, even after a medium change. This experiment is dependent on fluorescence intensity; therefore, it can be believed that the experiment would be more accurate to utilize the film-forming solution, where we could completely remove the treatment sample prior to imaging and not risk film remnants coating the coverslips.

Figure 5a displays microscope images of F-actin and DAPI staining on A375 cells. The presence of cells appears to dimmish over the course of treatment, as seen by the progression picture of F-actin and DAPI. However, as seen in Figure 5b, the fluorescence intensity of the individual cells selected at random does not decrease as excessively as may have been anticipated. Subsequently, the control group and both treatment groups were subject to having their overall fluorescence intensity measured by way of selecting three random locations on the coverslip and measuring the fluorescence given off by the entire group of cells that were pictured. The results can be seen in Figure 5c. The photographs obtained of DAPI staining were enhanced using ImageJ only after all calculations were performed. The purpose of the photos with enhanced contrast is strictly for visual comparison and they were not used for any obtaining of data.

### 3.6. HPLC Drug Release Profile

**Figure 6:**
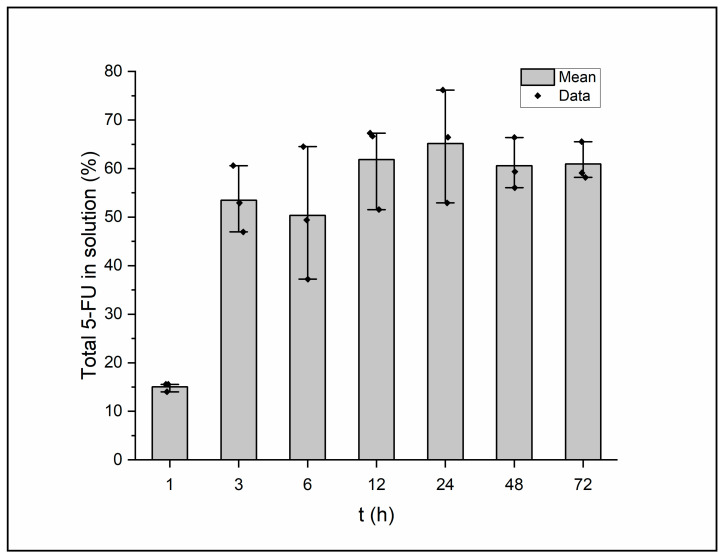
5-FU release rate over time from a complete EPS film immersed in PBS (*n* = 3). Statistical significance was determined with a one-way repeated measures ANOVA and Tukey’s test post hoc (*p* < 0.001).

This in vitro drug release assay provides data for a variety of variables involving the film, such as the nature of the drug of choice, compatibility between the polymer backbone and the drug, degree of crosslinking, and the chemical composition of the elution. A type of “sample-and-separate” method was employed to gauge the quantity of 5-FU release from the film’s structure over time [40]. The results of the assay can be seen in Figure 6. A burst release of the drug can be seen between the time points of 1 and 3 h. From here, the figure displays a controlled, maintained release of 5-FU through the remainder of the 72 h analysis time. Sethi et al. formulated and analyzed 5-fluorouracil loaded, glutaraldehyde-crosslinked chitosan nanoparticles and experienced a burst release within the first few hours of examination before the nanoparticles settled into a steady release for the 24 h measured [20]. Maximum release of 5-FU was seen to occur at the 24 h mark, where the samples averaged 65% of the total 5-FU released into the surroundings. The gap between the maximum drug amount released and the amount loaded could be explained by the high molecular weight of the EPS or a highly dense polymeric matrix. Diffusion of the drug from within the polymeric matrix will depend on the several aspects of the film including porosity, crosslinking density, the solubility of the drug, and the relationship between the polymer matrix and the drug [41]. Fluctuation of the polymer and crosslinker concentration could alter the extent of the drug release as well as solve the issue of the slight burst release the current film seems to exhibit.

### 3.7. Film Thickness

The thickness measurements for the EPS films containing 5-FU were determined to be 0.33, 0.35, 0.35, and 0.36 mm (*n* = 4), yielding an average of 0.35 mm ± 0.011. Indication of the lack of change and uniformity of the films following construction is possibly evident with the value calculated for standard deviation. One factor that can ultimately influence the measurement of thickness in this case would be proper mixing of all film components prior to casting, especially that of the EPSs. Incomplete mixing could lead to raised areas on the film’s surface that would result in inconsistent thickness around the film. It can be believed that a medicinal film or patch would benefit to exhibit maximum thinness without negatively impacting the mechanical properties. A slim and lightweight film delivering any therapeutic would allow for ease of being kept out of sight while wearing clothing. As is currently the case, the produced films seen in this preliminary formula do exhibit minimal thickness. Furthermore, it is theorized that by casting the same volume within a larger diameter evaporating dish, the current film-forming method presented here may yield thinner films that exhibit a more transparent body.

### 3.8. Swelling Ratio

The documented weights of the chemotherapeutic-loaded films are illustrated in Table 2. The 5-FU films were recorded as weighing between 1.129 g to 1.513 g. Disparities among the masses of the films may be due to a dense polymer matrix that leads to greater water retention or uneven thickness around a single film, as all films were measured at their center. The five films without 5-FU had an average weight of 1.022 g. The swelling capacity for each film when compared together differed to a large degree, being calculated as an average of 32.218% ± 15.

Swelling ratio explains the grade at which these films are crosslinked. If the value for swelling once the drug and crosslinker have been added is minimal, this is evidence of efficient crosslinking within the structure of the device. With the swelling ratio for these specific films having been recorded here, it is reasonable to next alter the crosslinker concentration and study the effects on the product.

## 4. Conclusions

This study presents a novel formulation for a chemotherapeutic-delivering exopolysaccharide film. Exopolysaccharides were produced from the thermophilic bacterium *Geobacillus* sp. WSUCF1, which breaks down unprocessed lignocellulosic biomass and utilize it as the primary carbon source for culturing, drastically reducing cost of production. In conclusion, a 5% EPS/5-FU was shown to be effective against human malignant melanoma A375 cells. After 12 h of A375 exposure to the contents of the film, cell viability results indicated near complete cell death. An LDH assay concluded similar results of potency against the melanoma, and further agreed with the results from the MTT assay with respect to the time-dependent toxicity. A drug release profile was acquired of a complete film allowed to soak in phosphate buffer saline. The aliquots extracted at several time points revealed a slight burst release of drug from the film between 1 and 3 h after the experiment began. A controlled and sustained release profile followed immediately after the burst release. Moreover, the film elution samples were not seen to contain greater than 65% of the initial 5-FU loaded into the film for the duration of the 72 h experiment. A tight and dense polymer matrix, assisted by crosslinker concentration, could be responsible for the lack of diffusion of the drug from the film’s structure. Future experiments could vary the concentrations of the polymer and crosslinker and examine the drug release profiles to optimize the performance of an EPS/5-FU film. A 5% wt/wt (5 g 5-FU/g EPS) may be unnecessarily potent for the size of the film and could potentially be reduced as to not waste drug or cause severe side effects when considering commercial applications. For now, the film’s formulation leaves it limited to topical delivery applications. However, the addition of permeation enhancers to the film’s formula could add to its diversity and pave the way for transdermal drug delivery purposes.

## Figures and Tables

**Table 1:** Variability in A375 cell density among exposure groups. Table values represent the quantity of cells that were manually counted from randomly chosen locations on the coverslips. The data are given as the mean accompanied by the standard deviation (*n* = 4).

	Untreated Group	12 Hours	24 Hours
Cell nuclei counted	57.3 ± 5.2	34.3 ± 3.7	20.3 ± 6.5

**Table 2:** The documented weights for films conjugated with and absent of 5-FU. The swelling ratio was quantified by representing initial film weight as the average weight of films vacant of 5-FU.

5FU-Loaded Film Weights (Grams)	5FU-Loaded Film Weights (Grams, Average)	Film Weights without 5-FU (Grams)	Film Weights without 5-FU (Grams, Average)	Swelling Ratio (%)	Swelling Ratio (%, Average)
1.211, 1.50, 1.402, 1.129, 1.513	1.351 ± 0.16	1.068, 1.087, 0.855, 0.981, 1.118	1.022 ± 0.095	18.516, 46.799, 37.209, 10.491, 48.072	32.218 ± 15

## Data Availability

Not Applicable.
